# Non-steroidal CYP17A1 Inhibitors: Discovery and Assessment

**DOI:** 10.1021/acs.jmedchem.3c00442

**Published:** 2023-05-16

**Authors:** Tomasz M. Wróbel, Flemming Steen Jørgensen, Amit V. Pandey, Angelika Grudzińska, Katyayani Sharma, Jibira Yakubu, Fredrik Björkling

**Affiliations:** † Department of Synthesis and Chemical Technology of Pharmaceutical Substances, Faculty of Pharmacy, Medical University of Lublin, Chodźki 4a, 20093 Lublin, Poland; ‡ Department of Drug Design and Pharmacology, Faculty of Health and Medical Sciences, University of Copenhagen, Universitetsparken 2, DK-2100 Copenhagen, Denmark; § Pediatric Endocrinology, Department of Pediatrics, University Children’s Hospital, Inselspital, Bern and Translational Hormone Research Program, Department of Biomedical Research, University of Bern, Freiburgstrasse 15, 3010 Bern, Switzerland

## Abstract

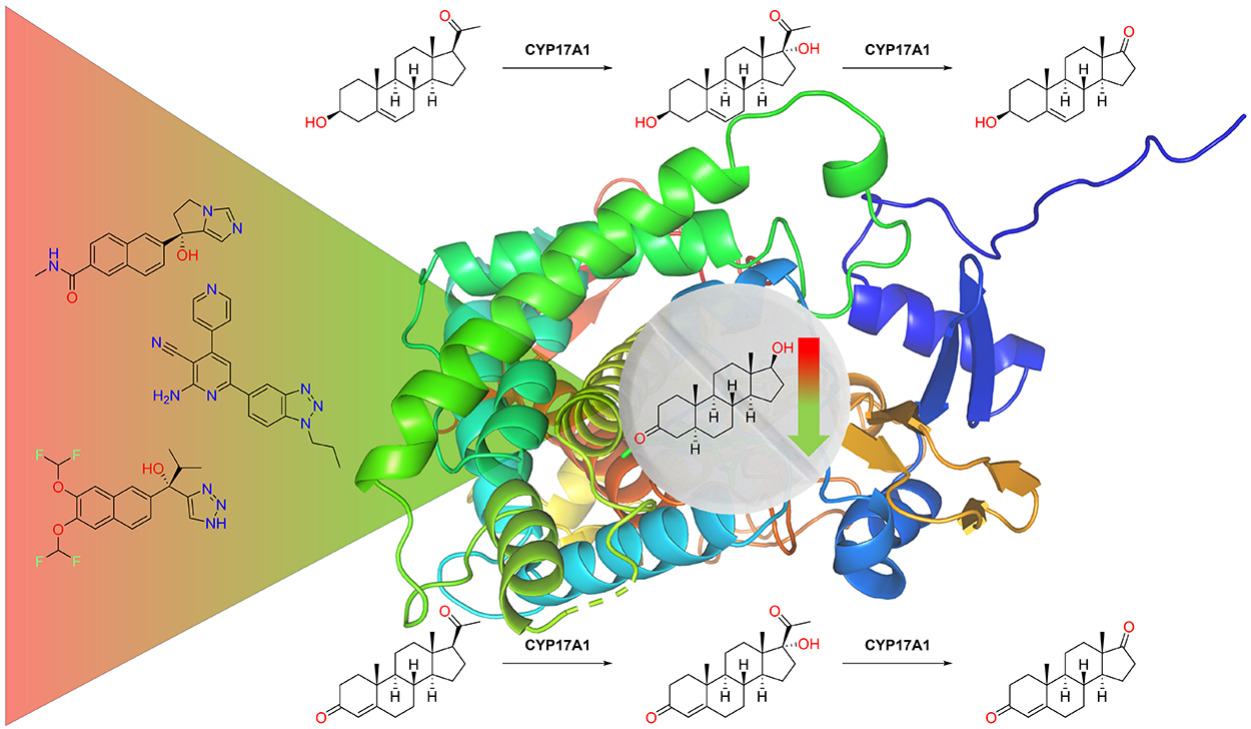

CYP17A1 is an enzyme that plays a major role in steroidogenesis and is critically involved in the biosynthesis of steroid hormones. Therefore, it remains an attractive target in several serious hormone-dependent cancer diseases, such as prostate cancer and breast cancer. The medicinal chemistry community has been committed to the discovery and development of CYP17A1 inhibitors for many years, particularly for the treatment of castration-resistant prostate cancer. The current Perspective reflects upon the discovery and evaluation of non-steroidal CYP17A1 inhibitors from a medicinal chemistry angle. Emphasis is placed on the structural aspects of the target, key learnings from the presented chemotypes, and design guidelines for future inhibitors.

## 1 Introduction

Cytochrome P450 17A1 (CYP17A1) is a membrane-bound dual-function monooxygenase belonging to the CYP 450 superfamily of enzymes. In humans, these proteins oxidize steroids, fatty acids, and xenobiotics and are crucial in steroid hormone biosynthesis and breakdown. Physiologically, CYP17A1 has an important role in the maturation and sex differentiation process, and the enzyme is found in the testes, adrenal glands, and ovaries. Furthermore, it contributes to the pathogenesis of diseases such as prostate cancer, polycystic ovary syndrome, and breast cancer.^1,2^ In view of this, extensive interest and effort have been put into the discovery of compounds that regulate the activity of CYP17A1, with one of the specific aims to find drugs useful in the treatment of castration-resistant prostate cancer.

CYP17A1 is encoded by a single gene on chromosome 10q24.3 and catalyzes two successive reactions, 17α-hydroxylation and 17,20-lyase transformation.^3^ The activity of CYP17A1 depends on redox interaction with P450 reductase (POR) and, in the case of the 17,20-lyase reaction, also cytochrome b5 (cyt *b*_5_).^4−6^

**Figure 1:**
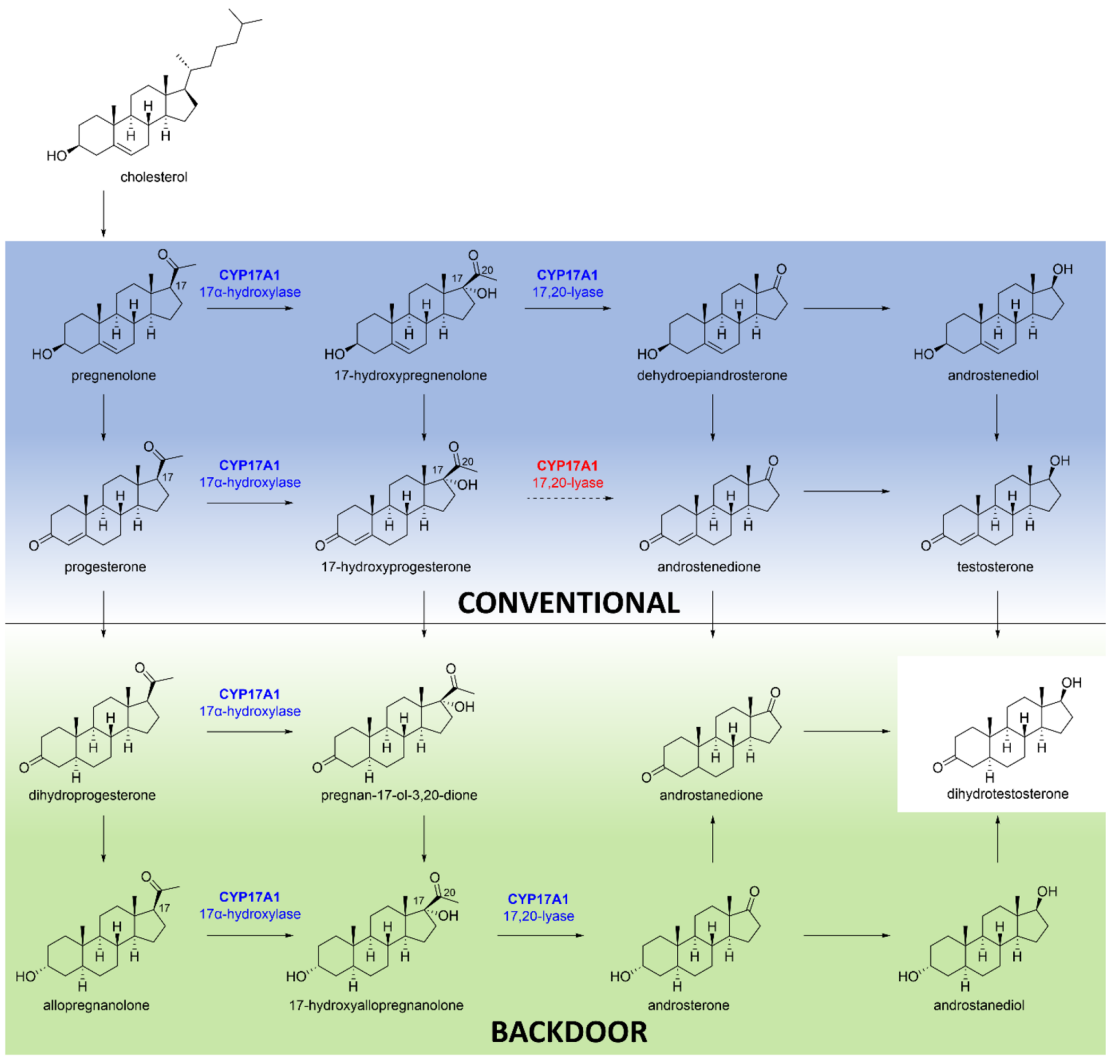
Androgenesis leading to the most potent androgen, dihydrotestosterone (DHT). “Conventional” and “backdoor” pathways are indicated with different color backgrounds. Red color represents inefficient catalysis in humans. Only transformations where CYP17A1 participates are labeled; other enzymes are omitted for clarity.

The lack of CYP17A1 activity results in a redirection of the synthesis towards the competing formation of aldosterone. The 17α-hydroxylase reaction hydroxylates both pregnenolone and progesterone at C17 to provide 17α-hydroxypregnenolone (17OH-Preg) and 17α-hydroxyprogesterone (17OH-Prog), respectively (Figure 1).^2,7,8^ Ultimately, the 17,20-lyase reaction breaks the bond between C17 and C20, transforming 17OH-Preg into dehydroepiandrosterone (DHEA) and 17OH-Prog into androstenedione. However, the direct conversion of 17OH-Prog to androstenedione is inefficient in humans, and androstenedione is formed primarily from the transformation of DHEA.^9^ 17OH-Prog is converted mainly to glucocorticoids, including cortisol.

Subsequently, DHEA and androstenedione are further transformed into testosterone, which is then converted to dihydrotestosterone (DHT). Androstenedione and testosterone also serve as substrates for estrogens. Besides the “conventional” pathway described above there is also the “backdoor” pathway, where a major androgen is an androsterone derived from 17-hydroxyallopregnanolone via the very efficient CYP17A1 17,20-lyase reaction.^10−12^ This pathway takes a detour around DHEA and androstenedione to produce DHT.^13,14^

The subtle differences between different CYPs and the preferred selectivity for the inhibition of the CYP17A1 lyase-catalyzed transformation have been addressed in structural and computational chemistry studies. Results of these investigations and compounds with increased selectivity have recently been reported which provide a promise for the next generations of CYP17A1 inhibitors.

To date, abiraterone acetate is the only CYP17A1 inhibitor approved for use in patients. This pioneering compound contains a steroidal scaffold similar to the endogenous CYP17A1 substrates. However, this drug is far from perfect. Side effects of abiraterone include vomiting, swelling, low potassium levels, high blood pressure, high glucose levels, joint pain, and diarrhea. In addition, adrenal insufficiency, liver failure, heart failure, arrhythmia, atrial fibrillation, and tachycardia are also possible side effects of abiraterone. These side effects stem largely from abiraterone promiscuity. At the molecular level, abiraterone is a potent inhibitor of CYP21A2 as well as CYP1A2, CYP2D6, CYP3A4, CYP2C8, and CYP2C9.^15,16^ CYP21A2 is responsible for production of glucocorticoids from progesterone and 17OH-Prog. To overcome the major side effect of steroid imbalance, abiraterone is now prescribed with corticosteroids like prednisone. In addition, abiraterone can be metabolized by HSD3B1 and 5α-reductase into 3-keto-5α-abiraterone that is capable of activating androgen receptor (AR), leading to the proliferation of cancer cells, undermining the therapeutic effects of abiraterone. Therefore, non-steroidal drugs that cannot be metabolized into androgens would be better candidates for androgen deprivation therapy (ADT), especially in individuals with higher expression or hyperactive variants of HSD3B1.^17^ The structural similarity of abiraterone to the substrates of other cytochrome P450 enzymes involved in steroidogenesis is one of the concerns with respect to selectivity and thus tentative side effects. Published work, covered herein, aims for novel non-steroidal compounds which may lead to both increased selectivity towards the CYP17A1 over other CYPs as well as inhibition of the CYP17A1 lyase activity versus the hydroxylase activity catalyzed by the same enzyme.

In this Perspective, we cover literature, from the earliest reports up to date, for compounds aimed to interact with and inhibit the CYP17A1 enzyme as a guide for further discovery and development of novel and important drugs in the field.

## 2 Role of CYP17A1 in Diseases

**Figure 2:**
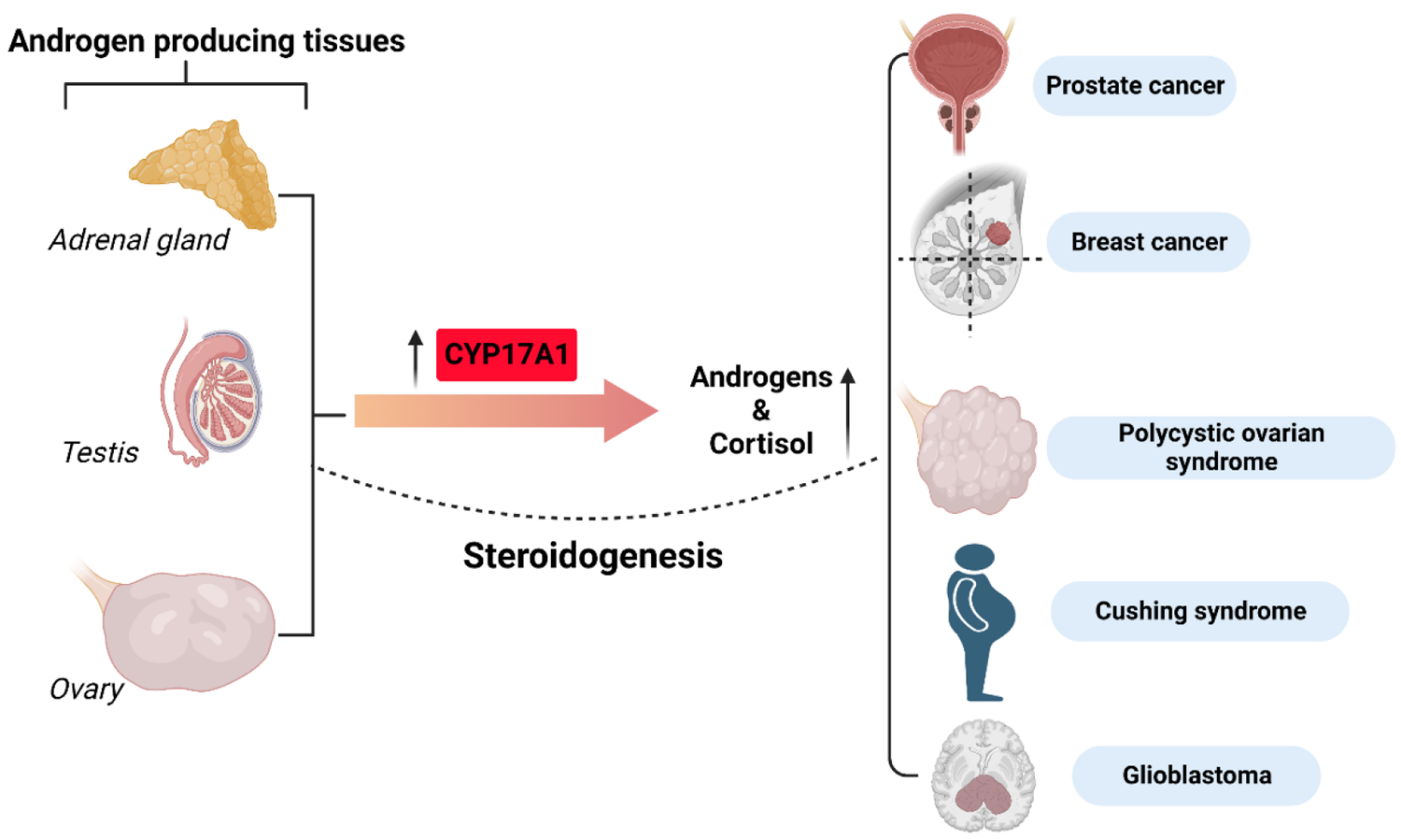
Summary of overexpression of CYP17A1 and activation in the production of steroid hormones linked to human diseases. Created with BioRender.com.

Due to its central role in regulation of steroids, changes in activities of CYP17A1 due to mutations or regulatory aspects may lead to multiple human disorders. Some of the human disorders linked to CYP17A1 are prostate cancer, polycystic ovary syndrome, breast cancer, Cushing’s syndrome, and glioblastoma (Figure 2). Additionally, links to many other disease conditions including hypertension, heart disease, Alzheimer’s disease, and leiomyoma have also been reported.^18−20^

### 2.1 Prostate Cancer

The major androgens implicated in the normal functioning of the prostate gland include DHEA, androstenedione, testosterone, and DHT. DHT is biologically the most active—it stimulates growth and maintains the morphology of the prostatic cells through interaction with the androgen receptor.^21^

**Figure 3:**
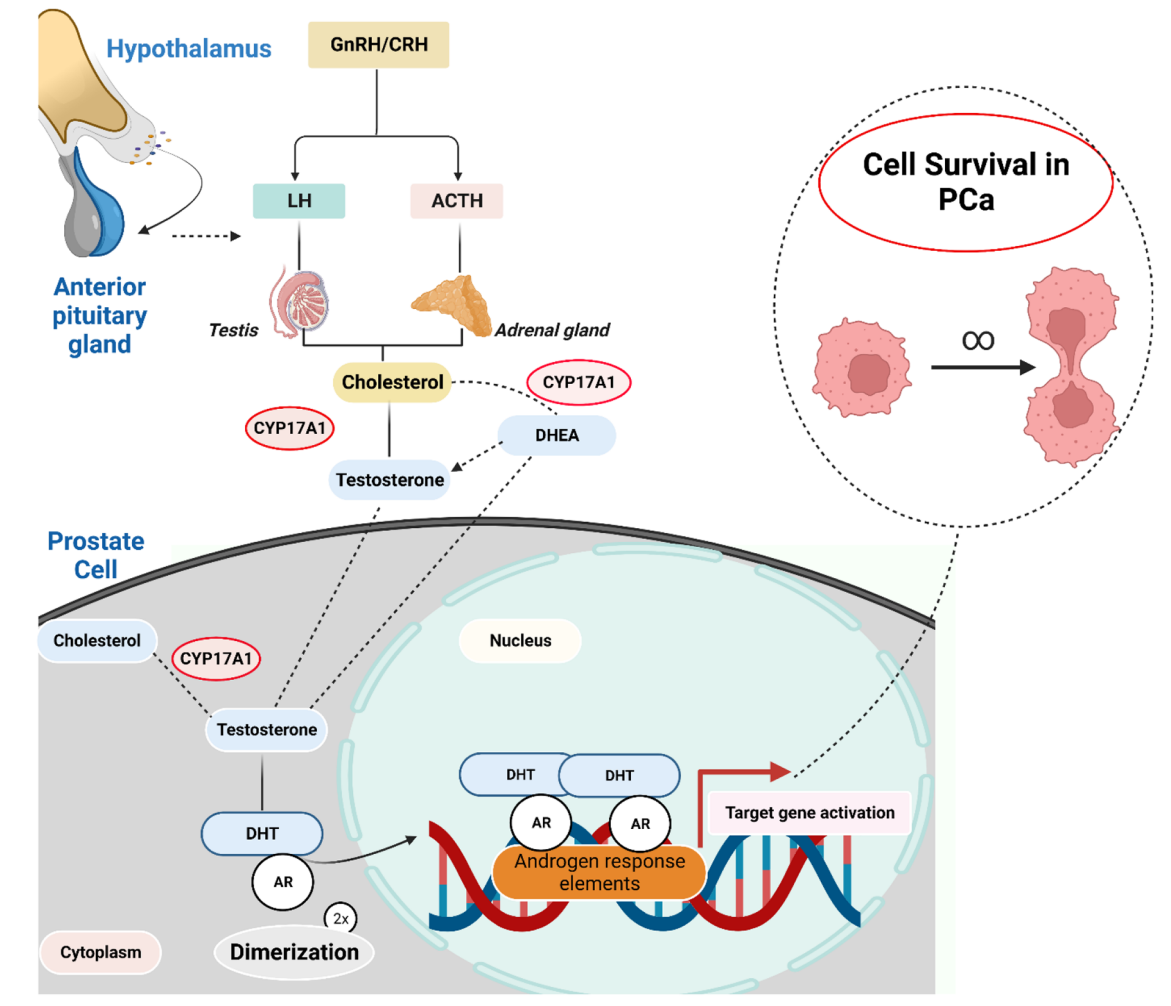
Androgen-dependent pathway in prostate cancer. The androgen receptor (AR, a hormone nuclear receptor) translocates into the nucleus upon activation by DHT as a homodimer and facilitates cell survival through the transcription of androgenic genes. Created with BioRender.com.

Androgens and their androgen receptors are considered key factors in the development of prostate cancer.^22^ This is confirmed by the positive response of patients to ADT. While initially the therapy brings the desired results, over time cancer cells acquire the ability to synthesize androgens *de novo*, and prostate cancer transforms into castration-resistant prostate cancer (CRPC).^23^ In this stage the hormone-dependent proliferation of the cells results from the great turnover of adrenal androgen precursors to testosterone, which is further reduced to DHT. CRPC is characterized by increased production of adrenal and intratumoral androgens, mutations, and increased expression of AR (Figure 3). Metastatic prostate cancer is manifested by a re-elevation of the prostate-specific antigen (PSA) marker despite the deficit of androgens and clinical deterioration.^24^ In the case of CRPC, a promising therapy is the use of inhibitors of the CYP17A1 enzyme, which is essential for the synthesis of androgens, by all routes of synthesis. So far, the only drug in use that represents this mechanism is abiraterone acetate. In 2011, it was approved by the FDA for the treatment of CRPC.

### 2.2 Breast Cancer

The transcription of genes that drive breast cancer is stimulated by estrogen-dependent signaling. It is believed that in some cases the androgen receptor replaces this signaling. Moreover, as shown by the androgen synthesis pathway, CYP17A1 indirectly plays a role in the synthesis of estrogens, the excessive signaling of which is associated with tumor development. Androgen receptor overexpression has been noted in some breast cancers.^25^ It was shown that the reduction of androgen levels was associated with the clinical improvement of patients.^26^ Inhibition of CYP17A1 appears to be a valid approach in the treatment of breast cancer.^27^

### 2.3 Polycystic Ovary Syndrome

Androgen excess is one of the clinical features of polycystic ovary syndrome (PCOS) and affects the development of the disease. CYP17A1 has been associated with PCOS and male pattern baldness.^28^ CYP17A1 is highly expressed in PCOS. In a study of Japanese women, age of menarche was significantly lower for women showing higher activities of CYP17A1.^29^ The excessive activation of PI3K/AKT signals occurring in the disease can lead to the excess of androgens and ovarian dysfunction.^1,30^

### 2.4 Cushing’s Syndrome

As CYP17A1 also regulates the synthesis of glucocorticoids, overexpression of the enzyme causes an overproduction of cortisol, an excess of which causes metabolic changes leading to Cushing’s syndrome. This is manifested by bone loss, high blood pressure, and type 2 diabetes. Inhibition of CYP17A1 lowers both androgen and cortisol levels, which is a promising handle in the development of a treatment for the disease.^31^

### 2.5 Glioblastoma

It has been reported that CYP17A1 is overexpressed in some forms of glioblastoma. DHEA plays a significant role here, as it protects cancer cells against apoptosis by reducing the effectiveness of chemotherapy. Inhibition of CYP17A1 results in the inhibition of DHEA production, which may be helpful.^32^ Following these assumptions, the effect of abiraterone on glioblastoma was investigated. Cellular assays and *in vivo* studies in mice models showed an inhibitory effect of the tested compounds.^33^

## 3 Structural Aspects

### 3.1 CYP17A1 Structure

**Figure 4:**
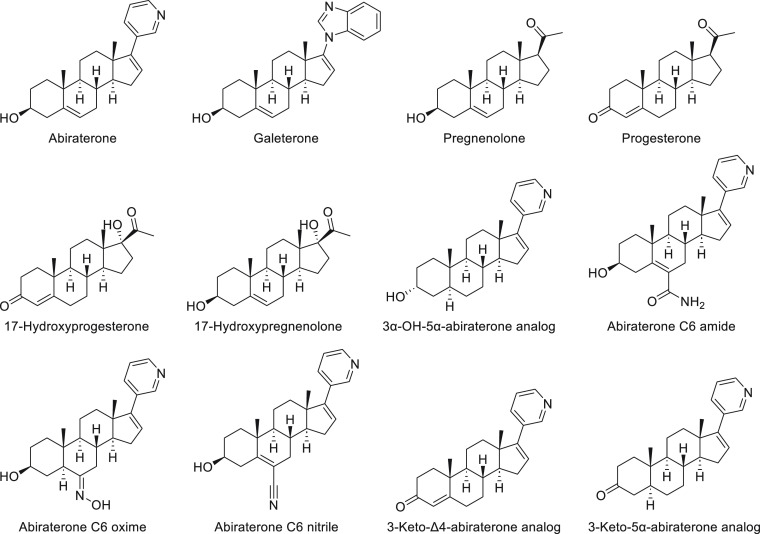
Structures of CYP17A1 steroidal ligands from the Protein Data Bank.

Initially, structural information was based on homology modeling,^34,35^ docking of natural substrates or synthetic analogues, and pharmacophore models.^36,37^ At present, a total of 16 structures of human CYP17A1 complexes are available from the Protein Data Bank (Table 1; for ligand structures see Figure 4 and Figure 19, below), comprising three different types of ligands (10 steroidal and 2 non-steroidal inhibitors and 4 substrates), each with their characteristic binding mode.

**Table 1:** List of Experimentally Determined Structures of Human CYP17A1 from the Protein Data Bank

**PDB**	**Ligand**	**Resolution [Å]**	**Notes**	**Year**	**Ref**
3RUK	abiraterone	2.6		2012	(38)
3SWZ	galeterone	2.4		2012	(38)
4NKV	abiraterone	2.6	A105L mutant	2014	(42)
4NKW	pregnenolone	2.5	A105L mutant	2014	(42)
4NKX	progesterone	2.8	A105L mutant	2014	(42)
4NKY	17α-hydroxyprogesterone	2.6	A105L mutant	2014	(42)
4NKZ	17α-hydroxypregnenolone	3.0	A105L mutant	2014	(42)
5IRQ	(±)-orteronel	2.2		2017	(44)
5IRV	VT-464	3.1		2017	(44)
5UYS	3α-OH-5α-abiraterone analog	2.4		2018	
6CHI	abiraterone C6 amide	2.7		2018	(43)
6CIR	abiraterone C6 oxime	2.6		2018	(43)
6CIZ	abiraterone C6 nitrile	2.6		2018	(43)
6WR0	3-keto-Δ4-abiraterone analog	2.7		2021	
6WR1	abiraterone	1.9	N52Y mutant	2021	
6WW0	3-keto-5α-abiraterone analog	2.0		2021	

**Figure 5:**
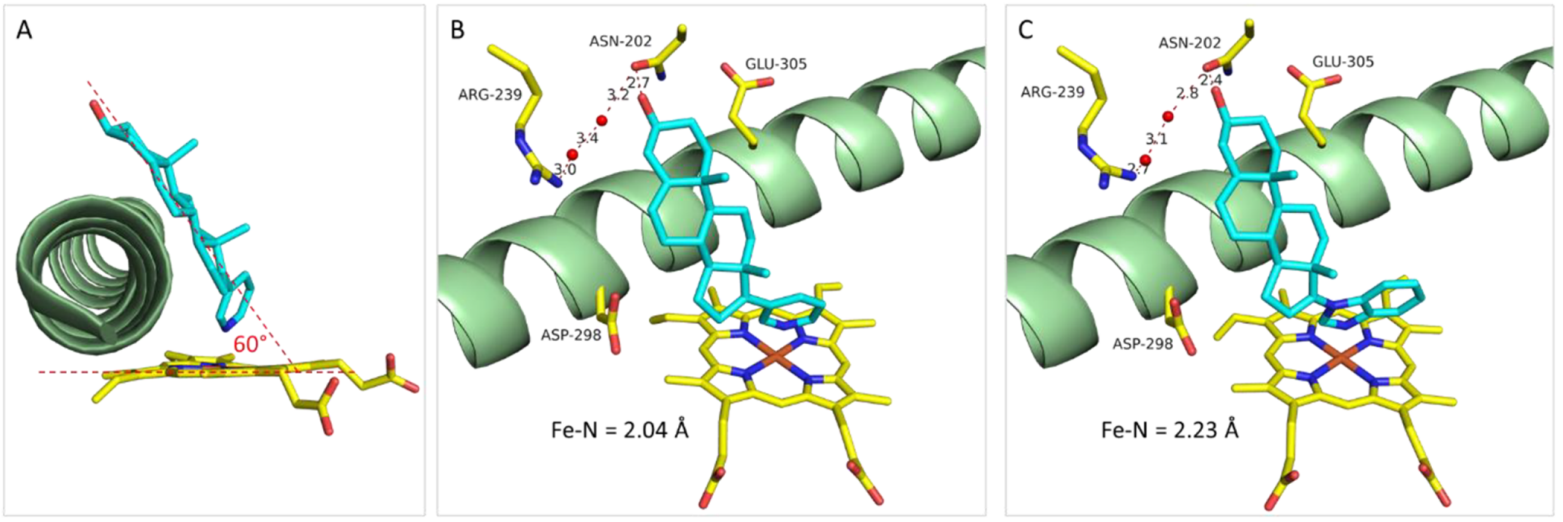
Binding modes of abiraterone (A, B) and galeterone (C) to CYP17A1 (PDB IDs: 3RUK and 3SWZ, respectively). For CYP17A1 the heme group and key residues are displayed as stick models and helix I as a green cartoon. Carbon, oxygen, nitrogen, and iron atoms are colored yellow, red, blue, and orange, respectively.

In 2012, the first crystal structures of CYP17A1 with abiraterone (PDB ID: 3RUK) and galeterone (PDB ID: 3SWZ) were published.^38^ Both structures show the enzyme-folding characteristic of CYP450 enzymes. The ligands assume a similar position to each other and, as previously predicted, interact with the heme iron through the sp^2^-hybridized nitrogen atom of pyridine or benzimidazole, creating a coordination bond. The steroid nuclei form an angle of 60° above the plane of the heme group, taking a position opposite the Helix I (Figure 5A). The 3β-OH group interacts with N202 in Helix F. The alpha surface of the steroid moiety is unsubstituted and flattens with respect to Helix I. This mode of binding differs from steroid binding in other cytochrome P450 enzymes. The C18 and C19 methyl groups are located between the B′ helix, the B4 loop, and the loop behind Helix F. Only three side chains of the ventricular wall are within 4 Å of either C18 or C19. The remaining wall of the pocket is filled with the hydrophobic side chains of A105, S106, A113, F114, I206, L209, V236, and V482.^38,39^

CYP17A1 has a hydrogen bond network at the top of the active site that interacts with abiraterone and galeterone, respectively. Residues N202 and R239 are forming hydrogen bonds, either directly or via water molecules, to the 3-OH group (Figure 5B,C).^38^ Two additional polar residues, E305 and D298, are also present in the active site. These residues do not interact with abiraterone or galeterone but are obvious targets for the design of non-steroidal inhibitors. These initial structures confirmed the expected interaction with the Fe in the heme group but also revealed important hydrogen-bonding and steric protein–ligand interactions, which have formed the basis for numerous theoretical studies on the mechanism of hydroxylase and lyase catalysis,^40^ recognition, and binding.^41^

**Figure 6:**
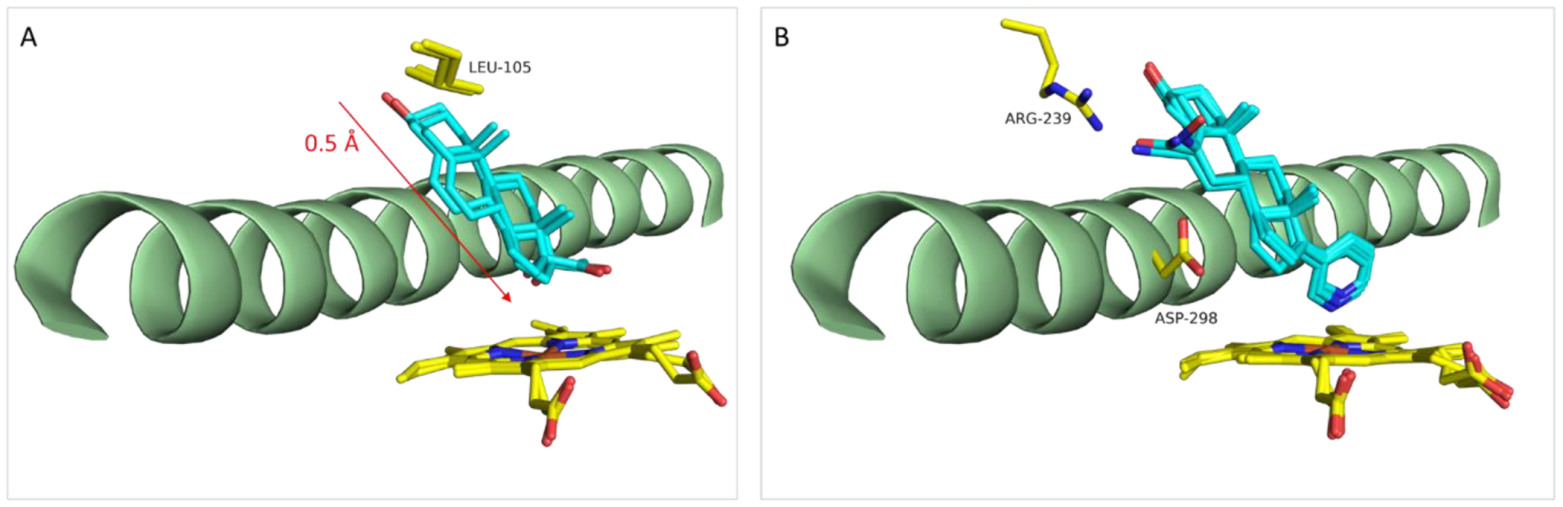
Two different binding modes of 17α-hydroxypregnenolone (A) and binding mode of the abiraterone analogs with C6 substituents (B) to CYP17A1. Color coding as in Figure 5.

The structures of the A105L mutant of CYP17A1 with a series of natural substrates for the 17α-hydroxylation and 17,20-lyase reactions (PDB IDs: 4NKV, 4NKW, 4NKX, 4NKY, 4NKZ) were published in 2014.^42^ The idea behind the A105L mutant was to modify the active site to resemble the 17,20-lyase reaction. The binding modes of the two 17α-hydroxylation substrates, pregnenolone and progesterone, and the two 17,20-lyase substrates, 17OH-Prog and 17OH-Preg, are nearly identical, but 17OH-Preg displayed two different binding modes in the crystal, one similar to the other substrates and one shifted 0.5 Å closer to the heme group (Figure 6A).^42^ The latter enabled the authors to explain the regioselectivity and substrate selectivity of the 17α-hydroxylation and 17,20-lyase reactions. Although these studies revealed important information on the mechanisms, it remains to be proven if the small structural differences imposed by the A105L mutation reflect the presence of the 17,20-lyase active-site conformation.

To improve the selectivity for CYP17A1 relative to CYP21A2, several abiraterone analogs were prepared with hydrogen-bonding substituents in the C6 position.^43^ The structures of three of the compounds (C6 nitrile, amide, and oxime, Table 1) revealed that the C6 substituent indeed was positioned between the two polar residues, R239 and D298 (Figure 6B). Experimental and computational studies showed that the increased CYP17A1 selectivity primarily was due to a reduced affinity for CYP21A2.^43^

The RMSD values based on the Cα atoms between the original abiraterone structure (PDB ID: 3RUK) and the other CYP17A1 structures in Table 1 are 0.33 ± 0.03 Å. This indicates that the CYP17A1 active site can accommodate structurally different ligands by changes in side-chain orientations without affecting the overall fold.

**Figure 7:**
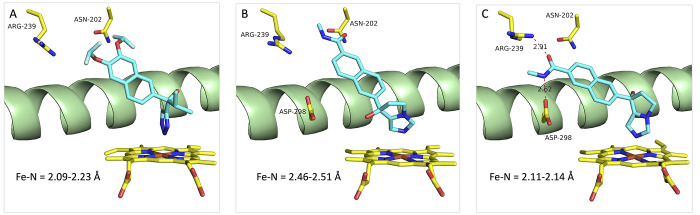
Binding mode of the non-steroidal inhibitors (*S*)-seviteronel (A) (PDB ID: 5IRV), (*R*)-orteronel (B), and (*S*)-orteronel (C) (PDB ID: 5IRQ) to CYP17A1. Color coding as in Figure 5.

It is worth mentioning that only two structures of CYP17A1 complexes with non-steroidal inhibitors, (±)-orteronel (TAK-700) and (*S*)-seviteronel (VT-464) (PDB IDs: 5IRQ and 5IRV respectively), have been published.^44^ Both compounds contain a naphthalene moiety substituted in the 2 position with a nitrogen-containing ring system. (*R*)-Orteronel and (*S*)-seviteronel bind “steroid-like” with the naphthalene ring occupying the same space in the CYP17A1 active site as the steroid part of the previously discussed steroidal inhibitors and the sp^2^-hybridized nitrogen atom in the substituent coordinating to the Fe atom in the heme group (Figure 7A and Figure 7B). Contrary to (*R*)-orteronel, binding of (*S*)-orteronel is tilted, enabling the substituent in the 6 position to form hydrogen bonds with R239 and D298 (Figure 7C). The Fe–N distance in (*R*)-orteronel is also substantial longer (2.5 Å) than the corresponding distances in (*S*)-orteronel and (*S*)-seviteronel (2.1 Å). An additional interesting feature with the orteronel structures, and to some extent also the seviteronel structures, is the presence of a peripheral binding site formed by different conformation of the loop between helix F and helix G. The function of this primarily hydrophobic site remains to be explored.^44^ The orteronel and seviteronel structures are interesting, as they may provide ideas for further optimization of lyase-selective non-steroidal inhibitors. The CYP17A1 structure, function, and therapeutic potential have also been reviewed.^45,46^

Considerable knowledge about the structure of CYP17A1 has also been derived from studying the clinical mutations typically found in patients with 17α-hydroxylase deficiency. Mutations R96W, R125Q, H373D/N, and R440H/C disrupt heme binding, resulting in a loss of enzyme activity. 17,20-Lyase activity is inhibited by mutations E305G, R347H/C, R358Q, and R449A.^47−50^

### 3.2 CYP17A1 Allosteric Site

The presence and utilization of an allosteric site in CYP17A1 is still a question for debate. It represents an until now not fully explored possibility for controlling the CYP17A1-mediated reactions. Potentially it opens the opportunity for imposing a conformational change of the CYP17A1 enzyme towards a lyase-relevant conformation, which would form the basis for the structure-based design of more lyase-selective CYP17A1 inhibitors.

Allosteric sites have been identified in other CYPs. Most of the studies have focused on CYP3A4, and it is proven without a doubt that this drug-metabolizing CYP contains an allosteric site. The structure showing that the fluorescent agent, fluorol, acts as an allosteric ligand and binds to a high-affinity binding site located in the substrate channel in CYP3A4 has recently been published.^51^

It has been known for many years that the 17,20-lyase activity of CYP17A1 relative to the 17α-hydroxylase activity can be stimulated by phosphorylation or by binding of cyt *b*_5_.^52^ Two mechanisms for the cyt *b*_5_ interaction with CYP17A1 have been proposed. Cyt *b*_5_ could either be responsible for supplying the second electron necessary to complete the catalytic cycle or be an allosteric modulator imposing a conformational change in CYP17A1.^53^ Indeed, cyt *b*_5_ is an electron donor, but it has been shown that the stimulation of CYP17A1 is an allosteric effect.^54,55^ Line broadening of the signals from certain residues in CYP17A1 was observed by means of NMR spectroscopy. This was interpreted as a function of cyt *b*_5_ binding causing a conformational change in CYP17A1. The effect was most pronounced for residues in the distal part of the CYP17A1 active site.^56^

A thermodynamic study of the energetics associated with the interactions between various CYPs and cyt *b*_5_ revealed that the CYP17A1-cyt *b*_5_ was enthalpy driven and that the interactions probably involved electrostatic interactions and formation of salt bridges and/or hydrogen bonds.^57^ This is consistent with previous observations that the anionic residues E48 and E49 in cyt *b*_5_ and the cationic residues R347, R358, and R449 in CYP17A1 are involved in the CYP17A1-cyt *b*_5_ interactions.^58,59^ NMR studies also revealed that cyt *b*_5_ combined with different substrates may impose different conformational states of the CYP17A1 structure.^60^ It is reasonable to assume that the cyt *b*_5_ residues involved in the interaction with CYP17A1 would provide a starting point for the design of peptides and peptidomimetics mimicking the allosteric effect of cyt *b*_5_ on CYP17A1. In a recent study the effect of a hendecapeptide EHPGGEEVLRE, comprising the above-mentioned E48 and E49 residues on CYP17A1, was investigated without obtaining the expected evidence for binding to CYP17A1.^50^

**Figure 8:**
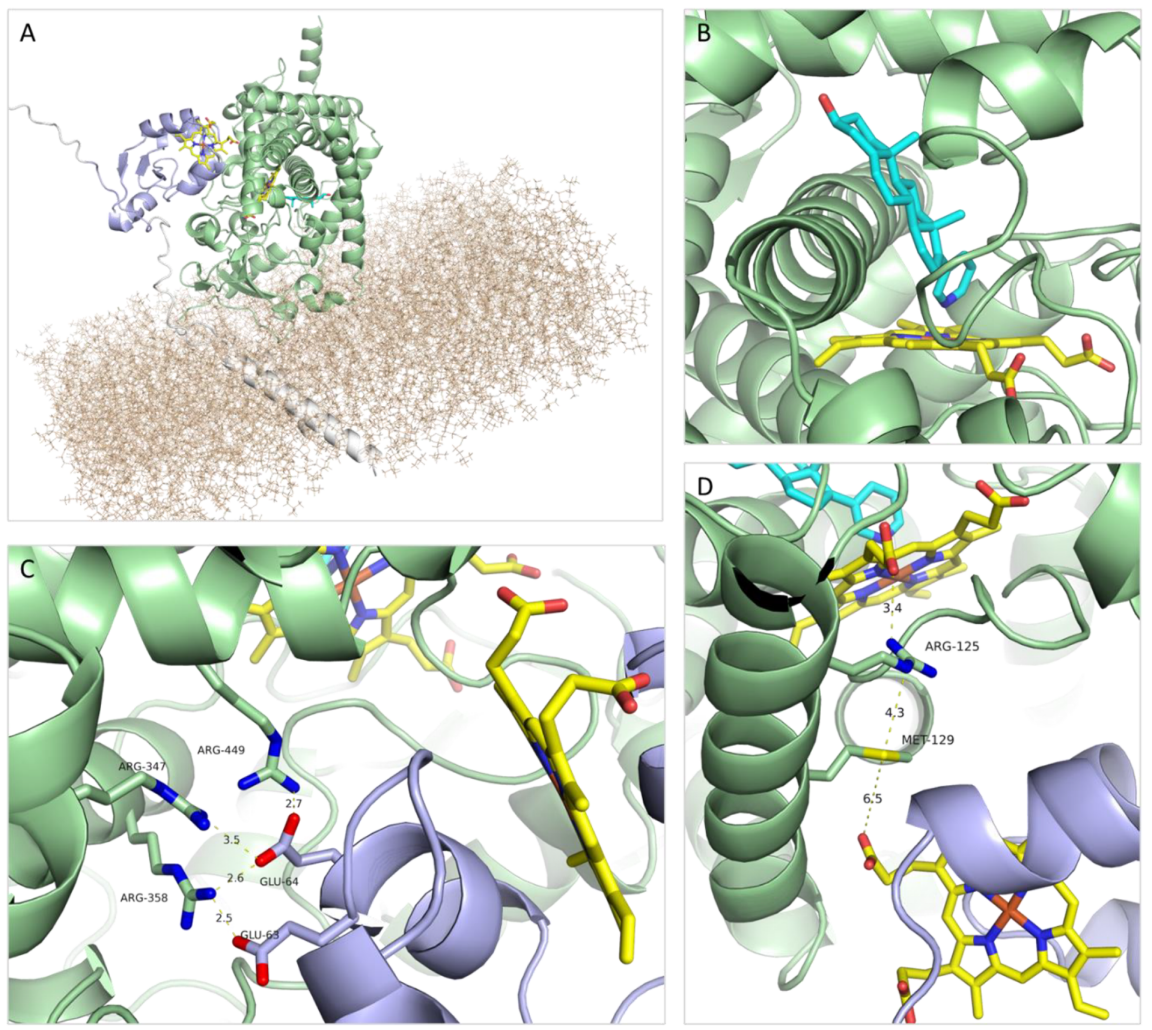
Three-dimensional model of the CYP17A1-cyt *b*_5_ complex anchored in the membrane. CYP17A1 green, cyt *b*_5_ blue, membrane beige, and anchoring helices and additional residues required for the AlphaFold modeling white (A). Close-up of abiraterone binding to the Fe atom in the heme group in the CYP17A1 active site (B). Key residues involved in the CYP17A1-cyt *b*_5_ binding (C). Residues proposed to be involved in the electron transfer from cyt *b*_5_ to CYP17A1 (D). Color coding as in Figure 5. For a movie illustrating the 3D relationships of the structure and interactions, see the SI.

To our knowledge no experimental structure of the CYP17A1-cyt *b*_5_ complex has been reported, but the CYP1A2-cyt *b*_5_ complex has been modeled by computational methods.^61^Figure 8 presents a model of the CYP17A1-cyt *b*_5_ complex constructed by the novel protein structure prediction method AlphaFold2 and subsequently embedded in a membrane analogous to the CYP1A2-cyt *b*_5_ model.^62^ We believe that such models may be useful not only to design novel compounds but also to help improve the understanding of other aspects of the enzymatic reactions, like the electron flow from cyt *b*_5_ to the CYP17A1.

## 4 Inhibitors

### 4.1 Brief Historical Overlook

One of the earliest reports goes back to the middle of the 20th century when dichlorodiphenyldichloroethane (DDD, TDE), a metabolite of DDT, was described as causing severe adrenal cortical atrophy in dogs.^63^ Since this discovery considerable effort was put into endocrine disruptors. However, no particular focus was placed on the inhibition of androgen production, and only single reports appeared on this topic.^64−69^

**Figure 9:**
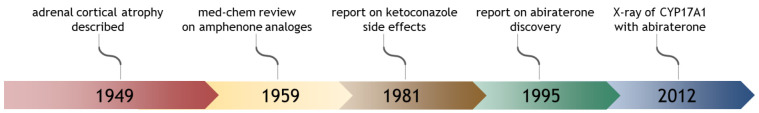
Timeline with milestones in CYP17A1 inhibitors discovery.

At the beginning of 1990 the pace of research into CYP17A1 inhibition picked up with the discovery of abiraterone, reported in 1995. This was fueled by a discovery a decade earlier of ketoconazole causing gynecomastia in men.^70^ Closer investigation of the underlying mechanism indicated that inhibition of the 17α-hydroxylation and 17,20-lyase reactions was responsible for this effect.^71^ The field gained increased interest when the first X-ray structure of CYP17A1 in complex with an inhibitor was reported in 2012 (Figure 9).^38^

### 4.2 Non-steroidal Inhibitors of CYP17A1

**Figure 10:**
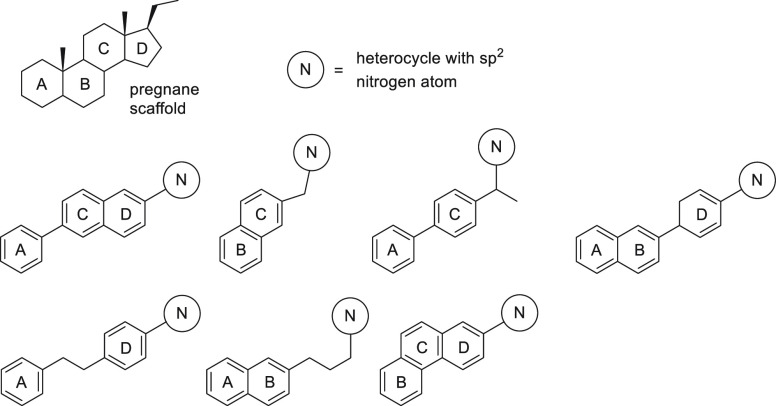
Different strategies aimed at mimicking the steroid scaffold commonly include deletion of one or two rings.

With a few exceptions, most of the medicinal chemist’s efforts rely on mimicking the steroidal scaffold of the natural CYP17A1 substrates (Figure 10). Keeping in mind that the critical moiety for enzyme inhibition is the lone pair of the sp^2^ nitrogen atom which interacts with the heme iron in CYP17A1, many designs emerged where a heterocycle was combined with a steroid mimetic ring system. These heterocycles included predominantly imidazole or pyridine, while other heterocyclic rings were less explored. Although the choice can include virtually any heteroaromatic ring containing nitrogen, imidazole and pyridine offer the most favorable binding energies.^72^ This section contains known inhibitors presented as different chemotypes. In some instances the choice is arbitrary as two chemotypes can overlap. It is important to remember that, when comparing IC_50_ values between compounds, care should be taken because these were often measured under different experimental conditions. In the case of CYP17A1 inhibition assays, the results are particularly sensitive to the substrate concentration and the ratio of CYP17A1:POR:cyt *b*_5_.^73^ In this regard, *K*_i_ is usually more informative than IC_50_ and repeated experimentation often reveals, for instance, the lack of initially reported selectivity.^44^

#### Azobenzene Derivatives

**Figure 11:**
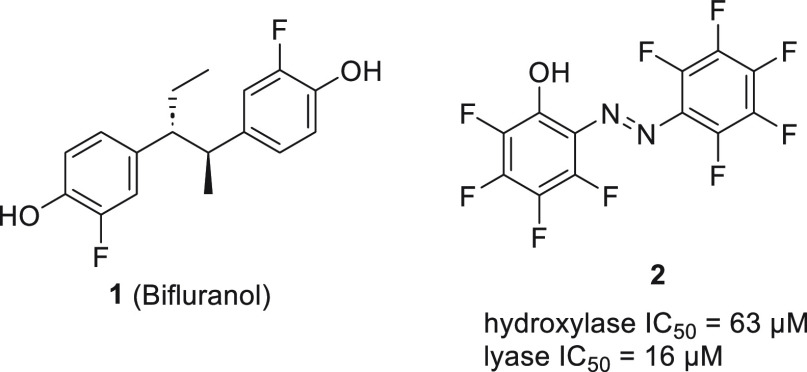
Representative CYP17A1 inhibitors of azobenzene analogs and bifluranol.

The discovery that bifluranol **1** inhibits CYP17A1 prompted the synthesis of its analogs.^74^ Compound **2** showed inhibition of CYP17A1 in the micromolar range (Figure 11). Although **2** achieved favorable selectivity of hydroxylase vs lyase 1:4, it was noted that the compound was unstable at the pH of the assay, forming dibenzoxadiazepine upon decomposition. Additional testing towards inhibition of 5α-reductase revealed no activity. These studies constitute one of the first attempts to create novel derivatives of a known inhibitor of CYP17A1.

#### Acetic Acid Derivatives

**Figure 12:**
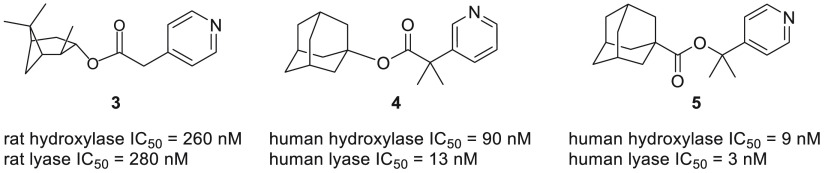
Representative CYP17 inhibitors from the acetic acid ester family.

A set of acetic acid esters was designed as inhibitors of aromatase.^75^ However, during testing these compounds showed significant CYP17A1 inhibition. Various alcohols were used for esterification, and closer analysis revealed improved potency with esters of bulkier alcohols (Figure 12). (+)-Isopinocampheol generated the most potent ester, **3**, while its enantiomer was less potent by an order of magnitude, showcasing the important role of stereochemistry. Unfortunately, these compounds, being esters, suffered from hydrolytic susceptibility. Also, the compounds displayed poor selectivity between hydroxylase and lyase inhibition. The problem of metabolic stability was addressed in the analogs with alkylated α carbons.^76^ Introduction of an alkyl group significantly increased resistance to esterases in rat liver microsomal preparations, with two groups being more effective than one regardless of their size. It was also noticed that this modification increased selectivity towards CYP17A1. Moreover, replacing the 4-pyridyl group with isomeric 3-pyridyl proved beneficial to the observed potency, as demonstrated by compound **4**. This modification also diminished activity towards aromatase. “Reverse esters”, with the reversal of the ester linkage, where the pyridine moiety resides on the alcohol part of the molecule, were designed to explore the effect of chirality adjacent to the pyridyl residue.^77^ This was done to circumvent racemization of enantiomers with a benzylic proton at the chiral center. The results showed dramatic differences in the inhibitory activity of monomethylated enantiomers of the 4-pyridyl series, while the enantiomers of the 3-pyridyl series were almost equipotent. The 4-pyridyl series was more potent than the 3-pyridyl in general, culminating in compound **5**, which had the best selectivity between hydroxylase and lyase and towards aromatase (Figure 12). In an attempt to improve the resistance to esterases, several amides analogs were prepared which displayed markedly decreased activity.

#### Phenyl Derivatives

**Figure 13:**
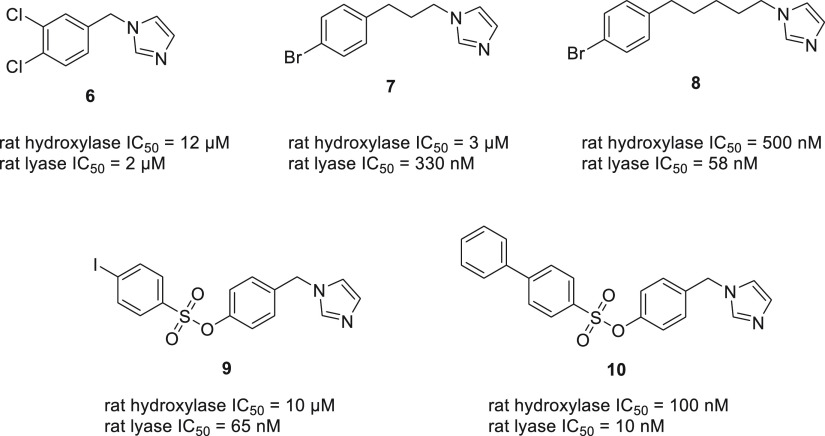
Representative CYP17 inhibitors of the phenyl derivatives group.

Phenyl derivatives constitute a case of extreme simplification, where the whole steroidomimetic scaffold has been reduced to just one aromatic ring connected with a carbon linker, varying in length, to a nitrogen-bearing heterocycle (Figure 13). Thus, the initial designs were aimed at exploring the effect of benzene substitution and the length of the linker.^78,79^

It was determined that the presence of a substituent in the phenyl ring was beneficial to the inhibitory activity, as was extension of the linker. No clear structure–activity relationship could be established in relation to the nature of a substituent, although disubstituted compounds were more potent than mono derivatives. However, a trend was observed in relation to the length of the linker showing potency increasing with the linker length, up to 10 carbons. Thus, an increased hydrophobicity led to increased potency.^80^ Compounds with the ethyl linker were unstable. Comparison between the incorporation of imidazole and triazole moieties demonstrated the former to be superior. The authors also tested their compounds against hydroxysteroid dehydrogenases and concluded the lack of specificity against these targets. The combination of the extended linker with different halogen atoms attached to the phenyl group was also investigated.^81^

It is noteworthy to add that in the majority of compounds it was possible to achieve a good selectivity profile between hydroxylase and lyase inhibition, as evidenced by compounds **6** to **8** (Figure 13). By adding a bulkier benzenesulfonate group it was possible to obtain compound **9**, which exhibited excellent selectivity of over 100-fold.^82^ Additional manipulation of benzenesulfonate moiety by changing the nature of the para-substituent did not improve the selectivity, although it produced compound **10** with enhanced potency.^83^

#### Stilbene Derivatives

**Figure 14:**
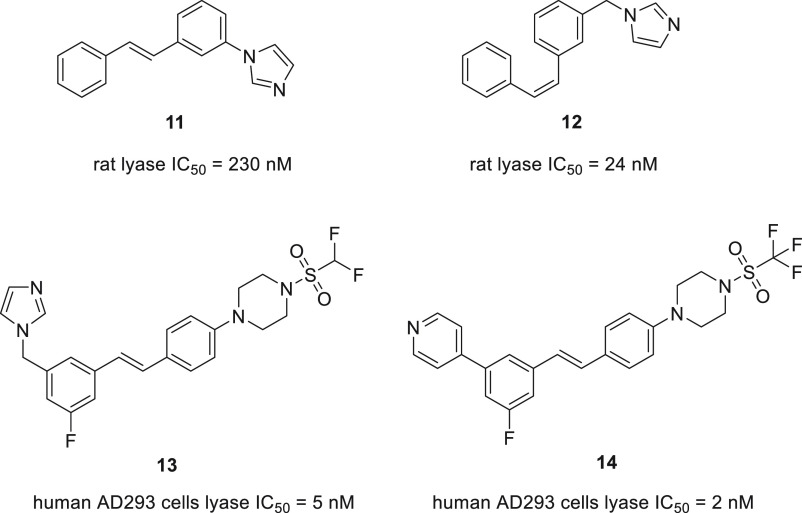
Representative stilbene-based CYP17 inhibitors.

The constrained nature of stilbene offers the possibility of constructing a steroid-mimicking ring system with a fixed geometry. The two geometrical isomers of stilbene (*E* and *Z*) have been used in compounds **11** and **12**. In this case, both the geometry of the double bond and the spacer linking imidazole with the scaffold played important roles in the reported activity (Figure 14). Additionally, compound **12** demonstrated *in vivo* an increased level of testosterone (135% of the control, measured at 5 h after treatment) despite an initial reduction. This was attributed to the cancellation of negative feedback caused by transient suppression of the testosterone levels.^84^

Compounds **13** and **14** were designed as stilbene-based derivatives of ketoconazole in a hope to overcome the synthetic limitation imposed by the complex ketoconazole core, thus enabling straightforward generation of analogs. A comparison between the energy-minimized structures suggested that the key binding components are similarly positioned. This mainly involved overlap of imidazole and pyridine heterocyclic moieties. Incorporation of the pyridine moiety gave more potent compounds in general compared to the imidazole moiety (Figure 14). Compound **14** displayed good selectivity for CYP17A1 against CYP19 (100-fold) and CYP3A4 (1000-fold).^85^ It is important to mention that numerous stilbene-based drugs are used in clinical practice. For example, tamoxifen is used to treat estrogen-receptor-positive breast cancer, and clomifene is used to induce ovulation. Both drugs interact with estrogen receptors, which engenders potential off-target liability for this class of compounds. Diethylstilbestrol, once widely used for prostate cancer treatment, no longer enjoys widespread use mainly due to cardiovascular toxicity caused by high doses.^86^

#### Analogs with a Heterocyclic Core

**Figure 15:**
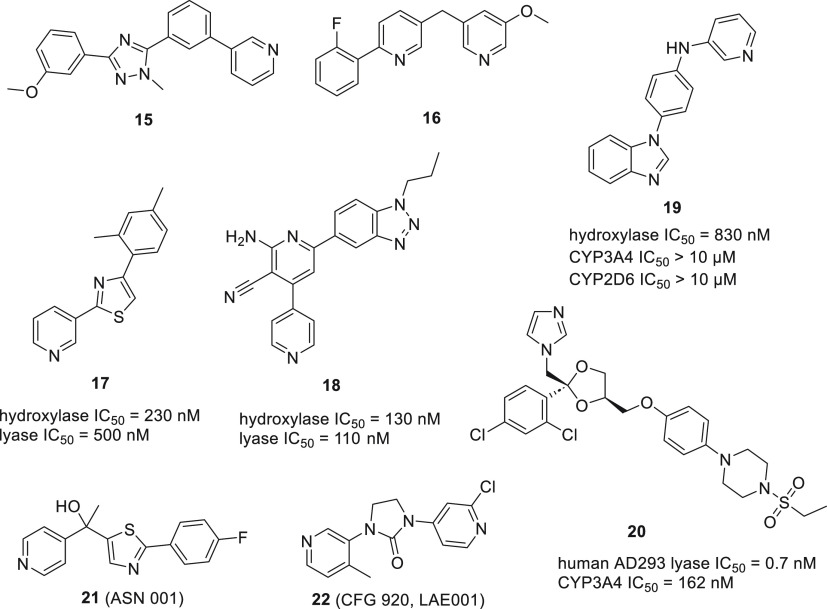
Representative inhibitors with heterocyclic cores.

In search for new inhibitors of CYP steroidogenic enzymes, compounds were designed using 1,2,4-triazole as a core structural element with a pyridine group responsible for interacting with the heme. It was reasoned that the triazole scaffold is a well-tolerated drug component and compared to a benzene core the triazole provides better solubility. Moreover, its basicity is lower than that of imidazole and the resulting geometry would better mimic the A and B rings of the natural substrate. While compound **15** was a nanomolar inhibitor of CYP11B1 and CYP11B2, none of the compounds showed inhibition of CYP17A1, and the structural analysis focused on selectivity between the above-mentioned enzymes (Figure 15).^87^

Compound **16** was designed for CYP11B1 inhibition as a wound healing agent. The compound was tested for selectivity vs CYP17A1 and was found to be a very weak inhibitor (5% at 2 μM) while CYP11B1 was potently inhibited (IC_50_ = 1 nM). No explanation for this selectivity was given based on structural analysis.^88^

Compounds with fragments of N-containing aromatic heterocycles, exhibiting the strongest interaction with the heme, were identified based on the binding energy calculations using density functional theory (DFT) methods. Compounds **17** and **18** were found as a result of this virtual screening. Both compounds displayed potent inhibition of CYP17A1 and good selectivity against CYP3A4, CYP2D6, and CYP21A2.^89^ Compound **19** was a result of linking two heterocyclic fragments present in abiraterone and galeterone.^90^ It displayed good IC_50_ and selectivity vs CYP3A4 and CYP2D6; however, further attempts to optimize this scaffold did not provide more effective compounds.^91^

Compound **20** represents an example of selective optimization of side activities (SOSA). This approach uses old drugs for new pharmacological targets.^92^ The obvious benefit would be a molecule with increased probability of having drug-like properties. It was reasoned that ketoconazole with its CYP17A1 “side activity” could be transformed into the entity possessing CYP17A1 as a “main activity” while diminishing other unwanted effects. Replacing the terminal acetyl group with the sulfonamide group resulted in increased potency towards CYP17A1 and markedly improved selectivity against CYP3A4 (Figure 15).^93^

Compound **21** (ASN001) was developed by Asana BioSciences as a selective CYP17A1 lyase inhibitor. However, no medicinal chemistry related papers could be found and only pharmacology data is readily available.^94^ Similarly, **22** (CFG920, LAE001) was initially developed by Novartis and licensed in 2017 to Laekna Therapeutics as a dual CYP17A1/CYP11B2 inhibitor.

**Figure 16:**
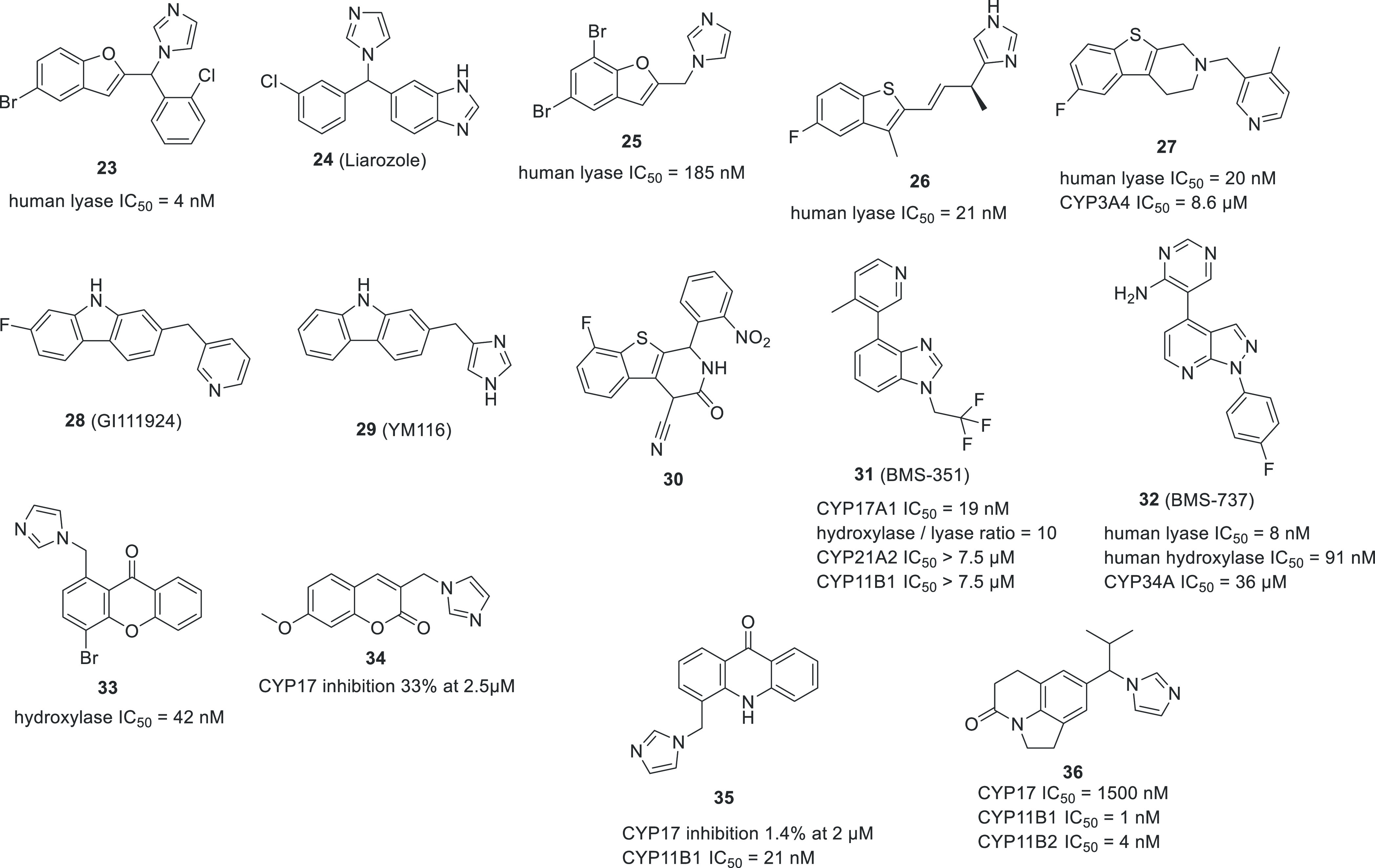
Representative inhibitors with fused 5- and 6-membered heterocyclic cores.

Attempts have been made at producing benzene-fused heterocycles. One of the examples is compound **23**, which was based on **24** (liarozole, R75251), a known inhibitor of several CYP enzymes. This compound replaces the benzimidazole moiety with benzofuran, retaining imidazole and chlorophenyl fragments (Figure 16).^95^ Further manipulation of the structure did not increase potency or selectivity, as exemplified by the truncated compound **25**.^96^

As a part of a drug discovery campaign, launched by Takeda, aimed at novel agents for the treatment of prostate cancer, multiple compounds were synthesized and tested. Benzothiophene derivative **26** was identified as a potent inhibitor.^84^ Notably this compound reduced testosterone levels in rats to 5% and 2% after 2 h and 5 h, respectively, demonstrating *in vivo* activity. Upon screening in-house compounds and inspecting modeling results, compound **27** was obtained.^97^ This compound bears resemblance to **28** (GI111924) and **29** (YM116) developed two decades earlier by GSK and Yamanouchi, respectively.^98^ The authors reasoned that the substitution of nitrogen for a sulfur atom in tetrahydro-β-carboline will better accommodate the molecule in the enzyme binding pocket. In addition, introduction of a substituent in the pyridine ring provided a compound with a better fit of this moiety. As a result, **27** proved to be a potent inhibitor with marked selectivity against other CYP isoforms and good *in vivo* activity measured as rat serum testosterone level. Interestingly, based on those findings another group designed and tested benzothiophene analogues, e.g., compound **30**, where an additional phenyl ring bears the nitro group which is claimed to be responsible for the interaction with the heme.^99^

Researchers at Bristol-Myers Squibb found a potent indazole derivative while screening their internal compound collection.^100^ They set out to identify a chemotype allowing for continuous CYP17A1 inhibition, reasoning it would be required for efficacy. By changing the indazole scaffold to benzimidazole they were able to identify compound **31** (BMS-351) with potent inhibition of CYP17A1 and enhanced metabolic stability. Moreover, **31** demonstrated higher lyase/hydroxylase selectivity compared to abiraterone, which was attributed to its reversible nature. A superior steroidal profile with >90% decrease in testosterone in cynomolgus monkeys was achieved together with minimal disruption to progesterone and cortisol levels. In further attempts to optimize the desired properties, a wide range of alterations to the scaffold as well as to the heme binding moiety were explored, culminating in compound **32** (BMS-737) with good selectivity against various CYPs and potency and efficacy similar to **31** (Figure 16).^101^

Several fused six-membered heterocyclic compounds were described mainly as CYP19A1 (aromatase) or CYP11B1 and CYP11B2 inhibitors. Most of the reported chromone and xanthone derivatives displayed weak activity towards CYP17A1 with few exceptions, like compound **33**.^102^ It is noteworthy to add that the authors used comparative molecular field analysis (CoMFA) to design these compounds. Coumarin derivatives were selective towards CYP19A1 and exhibited only low CYP17A1 inhibition, as exemplified by **34**.^103^ In a similar fashion, compounds based on a quinolinone scaffold were weak CYP17A1 inhibitors. Compound **35** was only able to inhibit CYP17A1 by 1.4% at 2 μM, and **36** inhibited CYP17A1 with IC_50_ = 1.5 μM (Figure 16).^104,105^ In order to understand the observed selectivity, the authors used sequence alignment between the four CYP enzymes (CYP17A1, CYP19, CYP11B1, and CYP11B2).^104^ While not specifically designed to target CYP17A1, these compounds provide useful information on selectivity vs other CYPs.

#### Diphenylmethane Derivatives

**Figure 17:**
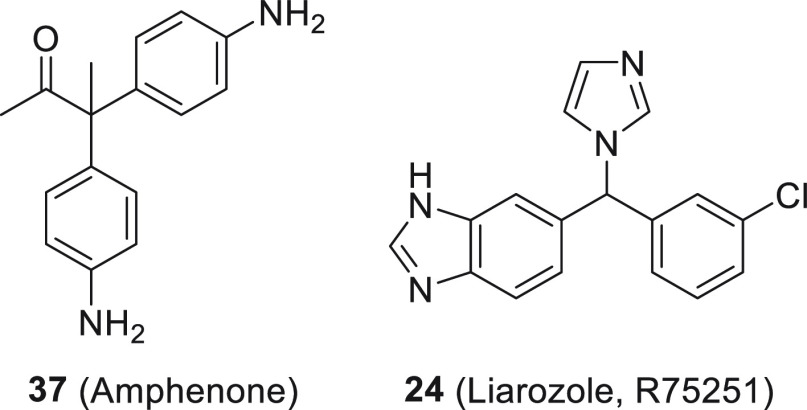
Examples of diphenylmethane analogs.

Amphenone (**37**, Figure 17) and its analogs belong to the earliest substances found to inhibit steroidogenesis.^64^ It was tested in humans as a treatment for adrenocortical carcinoma. The effects on androgens were limited with this compound. Higher activity was observed for compound **24** (liarozole, R75251), which was capable of reducing testosterone plasma levels to castrate levels in male dogs.^106^ Similar results were obtained in humans.^107^ Amphenone did not reach clinical practice while liarozole received orphan drug designation for the treatment of congenital ichthyosis. Liarozole is also capable of CYP26A1 inhibition and has been used as a tool compound.^108^

#### Biphenyl Derivatives

**Figure 18:**
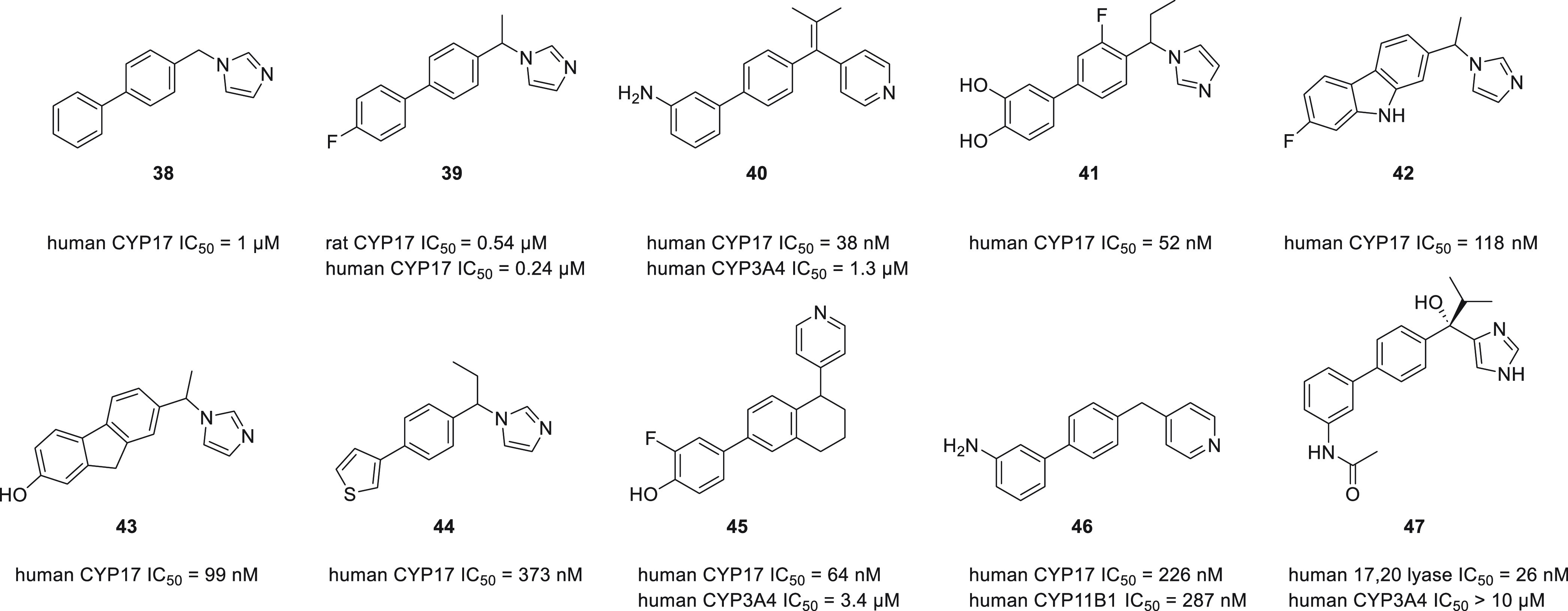
Examples of biphenyl inhibitors.

The biphenyl moiety represents a widely explored possibility to mimic A and C rings of the pregnane scaffold (Figure 10). *In vitro* studies have shown that 3-imidazol-1-yl-methyl-substituted compound **38** and its derivatives are particularly notable (Figure 18).^84,109^ However, these compounds lacked sufficient activity *in vivo*. It was assumed that this was related to their fast metabolism.^110^ In order to slow down phase 1 metabolism, a series of polyfluorinated compounds was designed.^111^ The location of the fluorine atoms turned out to be important because the meta-substituted compound was more resistant to biodegradation than the ortho-substituted one. It has been shown that fluorine in position 3 contributes to both a stronger interaction with the active site and an increase in metabolic stability, consequently increasing the half-life of the compound.^112^ Introduction of a fluorine atom is a common strategy employed by medicinal chemists. This can influence the metabolic stability or facilitate cell membrane permeation of a molecule.^113^

Modifications, including introduction of various substituents and structure rigidification, have been carried out to improve activity.^114^ It was found that introduction of fluorine into the distal aromatic ring and a methyl group into the methylene bridge connecting the biphenyl moiety and imidazole brought an increase in potency as well as sustained reduced plasma testosterone concentration, as demonstrated by compound **39**. In subsequent years it was determined that the single group on the methylene bridge can be the key to potency and selectivity among several CYP enzymes.^115^ Compound **40** displayed potent inhibition of CYP17A1. It has also been reported that substitution of the A ring with polar substituents leads to strong inhibitors, represented by compound **41**.^116^ Another study showed that the activity of the compounds could also be increased by constraining the molecule in the form of carbazole **42** or fluorene **43**. However, these compounds required further optimization for improved CYP17A1 selectivity.^117^

Another strategy aimed at improving the potency was replacing the A or C aromatic nuclei with different heterocycles. Compound **44** was a potent inhibitor and showed a longer duration of action *in vivo* than the reference abiraterone.^118^ Attempts have been made to utilize the ACD and ABD (Figure 10) strategies and annulate A or C rings.^119,120^ However, these compounds did not display significant CYP17A1 inhibition. Further studies showed that a potential strategy to improve the activity involved dearomatizing ring D of the ACD system. This led to the potent and selective CYP17A1 inhibitor **45**.^121^

Further modifications of the biaryl compounds led to design of dual inhibitors of CYP17A1 and CYP11B1. The new strategy was to contain elements of the pharmacophores derived from abiraterone as an inhibitor of CYP17A1 and metyrapone as an inhibitor of CYP11B1. A pyridyl group was used in place of an imidazole group, resulting in a dual inhibitor **46**.^122^ The importance of the chirality at the linker position was highlighted by compound **(−)-47** which was a very potent CYP17A1 inhibitor and had excellent selectivity for CYP3A4 (>300-fold). The dextrorotatory enantiomer was over 10 times less potent (IC_50_ = 340 nM vs 26 nM). Moreover, the compound displayed a sustained decrease in serum testosterone levels after single oral dosing.^123^

#### Naphthalenes

**Figure 19:**
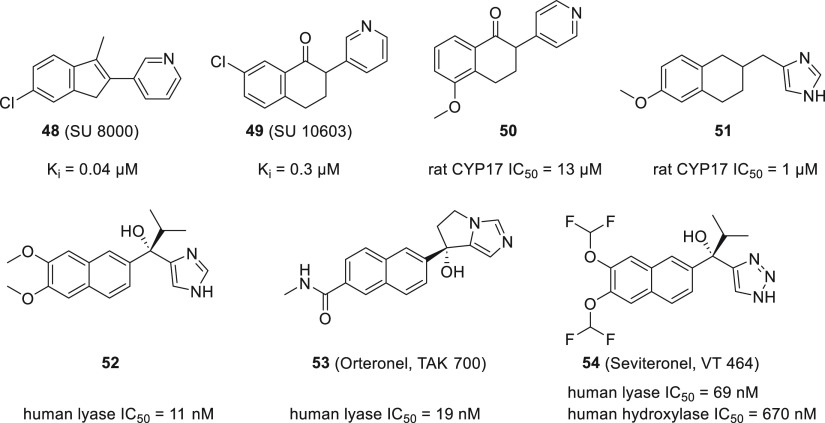
Representatives of naphthalene CYP17A1 inhibitors.

Based on two well-known CYP17A1 inhibitors **48** (SU 8000) and **49** (SU 10603), a series of indanes and tetralines were designed, out of which compound **50** was the most potent (Figure 19).^124,125^ Further modifications included scaffold hopping, utilizing various heme-binding heterocycles, and modifying the linker between tetraline and heterocyclic moiety into a fused cyclopropane ring or imidazole ring.^126−131^ In subsequent research it was determined that the presence of a tetralone oxo group was not essential, but it was beneficial to add a hydrogen bond acceptor, presumably mimicking the natural substrate. Compound **51** showed potent CYP17A1 inhibition and good selectivity.^132^ Attempts have been made to introduce an element of unsaturation into the tetralin ring as well as an oxime group into the side chain. These modifications yielded only marginal or no inhibitory properties when tested in human CYP17A1.^133−135^

Another modification of the naphthalene derivative **51** involved inclusion of a hydroxyl and an isopropyl group at the methylene bridge. This was crucial for limiting the effect of compounds on liver enzymes. However, the best results were achieved by introducing additional methoxy groups in the 6 and 7 positions, which resulted in compound **52**.^136^ This compound also proved effective in *in vivo* studies in the monkey model. Subsequent manipulation of the naphthalene substituents resulted in tricyclic derivatives and eventually in **(+)-53** (orteronel, TAK-700).^137,138^ Orteronel demonstrated potent reductions in serum testosterone and DHEA concentrations after single oral dosing (1 mg/kg) in cynomolgus monkeys. Selectivity for CYP17 over other CYP enzymes was attributed to the conformational rigidity and low clogP value. Similar to orteronel, another orally active compound, **(−)-54** (seviteronel, VT-464), was designed and proved to be a potent and selective CYP17A1 inhibitor with substantial *in vivo* activity.^139^ However, the reported selectivity was not replicated under the very strict conditions.^44^ Orteronel and seviteronel represent a handful of non-steroidal inhibitors reaching clinical trials; however, they did not succeed to reach clinical practice.

#### Natural Products

**Figure 20:**
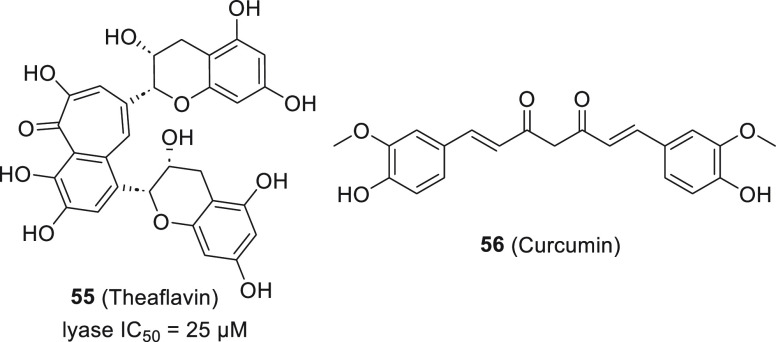
Representative inhibitors belonging to natural compounds.

Natural products represent a unique chemotype because they do not possess a nitrogen atom while the vast majority of the known CYP17A1 inhibitors do. During the screening for CYP17A1 inhibitors, potent activity of methanol extracts from green and black tea was found.^140^ These fractions are rich in catechins. Detailed studies on commercially acquired various catechins and theaflavins revealed inhibitory activity surpassing that of ketoconazole. Theaflavin **55** displayed IC_50_ = 25 μM for the lyase reaction (ketoconazole IC_50_ = 35 μM). Similarly, turmeric extract containing curcuminoids was found to inhibit CYP17A1 (Figure 20).^141^ Curcumin **56** docked to the 3RUK model showed a resemblance to the steroid substrates with phenolic oxygen distanced 2.4 Å from the heme iron. These compounds seem to offer an interesting starting point for further optimization.

## 5 Assays

### 5.1 NCI H295R Cell Model

The current standard model system to study molecular and biochemical mechanisms of steroidogenesis is the NCI H295R cell line.^142^ This cell line was established from a series of strains of adrenocortical carcinoma tumor cells obtained from a 48-year-old black female exhibiting conditions like acne, facial hirsutism, diarrhea, weight loss, edema, and abnormal menses.^143^ The initial cell line, NCI H295, was further developed into the NCI H295R strain having a shorter doubling time, adherent monolayer growth, and retained steroidogenic capacity over subsequent passages. The adrenal gland is a complex system, divided into specific zones of differentially expressed genes involved in the production and regulation of steroids. Cell models arising from animal or human tissues require zone-specific primary regulators to facilitate steroid production, and the steroid profile often changes with successive passaging, response factors, and growth conditions. NCI H295R cells express genes from all three zones of the adrenal cortex, providing an excellent system that closely reflects human adrenal physiology. The available mouse adrenocortical cell line, Y1, produces mainly glucocorticoids and mineralocorticoids and cannot express genes involved in the production of sex steroids, rendering them inefficient to study the production of androgens and CYP17A1 activity.^144^ Studies with cDNA isolated from hamster adrenal libraries showed a preference for the Δ4 pathway to produce DHEA like the human adrenal system. Unlike the mouse model, it might serve as a better animal model to study human steroid metabolism.^145^ So far, among all these systems, the NCI H295R cell line is the preferred model as it is more convenient, cheaper, and robust to perform enzyme kinetic studies and molecular biology-based assays.

### 5.2 Enzyme Assays

One of the oldest methods to analyze enzyme activity is the use of colorimetric assays to detect androgens in urine samples.^146^ Major disadvantages are the requirement of large sample volumes, lack of specificity, and poor range of detection. The earliest enzyme assays to investigate the production of androgens were performed in microsomes isolated from testicular tissue extracts from guinea pigs which were found to be oxidizing progesterone to testosterone and acetic acid. The products formed were detected using radioactive substrates labeled with carbon-14 or tritium at specific positions.^147^ Different isolation and separation techniques were adopted to quantify the steroids present in the reaction mixture, including methods like direct distillation, paper chromatography, or thin-layer chromatography (TLC) using different organic solvent systems as mobile phase.^148,149^ These methods, combined with radioimmunoassay (RIA) or protein binding assay, enabled the quantitative detection of steroids with enhanced specificity and sensitivity.^150−152^ However, it has a few drawbacks such as the handling of radioactive materials, the requirement of intensive labor work, and cross-reactivity with other steroids resulting in the detection of unwanted steroids in a complex sample.^153^ With the development of techniques like normal-phase high-performance liquid chromatography (HPLC), separation and detection of both C19 and C21 steroids became possible and enabled the assay of CYP17A1 activity from both Δ4 (progesterone) and Δ5 (pregnenolone) pathways. Typical chromatographic separation is carried out over a hexane–tetrahydrofuran gradient system with a silica stationary phase. The flow system is coupled to a flow cell radioactivity detector sensitive to tritium. The use of radiolabeled substrates eliminates the need for internal standards and increases the sensitivity for detection.^154,155^ Simultaneously, microsomal-like systems containing recombinant and purified CYP17A1 were developed to study the effect of mutant CYP17A1 proteins on enzyme activity and overall steroid metabolism with respect to wild type.^156−158^ Combining the advanced techniques of gas chromatography (GC) and HPLC with sophisticated detection systems utilizing mass spectrometry (MS), methods like GC-MS and LC-MS/MS were developed and became widely adopted for performing whole steroid profiling in different biological samples.^159−162^ With the advancement leading to decreased sample volume requirements and detection of a range of steroids present at even lower concentrations, most of the shortcomings of the previous techniques were resolved. GC/LC-MS/MS has proven to be beneficial to generate steroid profiles from different types of assays as well as diagnostics/patient samples for various clinical diagnoses in a faster and more efficient way.^163−165^ Although it has several advantages, it is comparatively expensive and demands highly trained professionals to operate the instruments and analyze the data. This renders it less suitable to perform large-scale drug screening of small molecule inhibitors to target CYP17A1 activity. In this case, the preferred technique is the separation of radiolabeled steroids by TLC and quantification using autoradiography or direct measurement of radioactivity with the help of a liquid scintillation counter.^16,141,166^

## 6 Perspective

### 6.1 Desired Inhibitory Profile

The CYP17A1 enzyme is a key catalyst involved in steroidogenesis, thus the biosynthesis of steroid hormones in the adrenal glands, gonads, and placenta. CYP17A1 inhibitors can be used to treat a range of medical conditions, including hormonal imbalances or endocrine-dependent cancers. From a drug discovery perspective, the desired inhibitory profile of CYP17A1 inhibitors depends on the specific disease being targeted. For example, in the treatment of prostate cancer, the goal is to reduce androgen synthesis, so a highly specific and potent inhibitor of CYP17A1 would be ideal. On the other hand, in the treatment of congenital adrenal hyperplasia, a less potent inhibitor that does not completely shut down steroidogenesis may be preferred to avoid significant hormonal imbalances. To avoid undesired side effects a high specificity towards the target CYP17A1 cytochrome is important. To address this issue the subtle structural differences in the different CYPs need to be taken into account during the medicinal chemistry optimization. In terms of general side effects that can be tolerated, this will depend on the specific medical condition being targeted. For example, in the treatment of prostate cancer, some of the common side effects associated with CYP17A1 inhibition include hot flashes, osteoporosis, and decreased libido. These side effects may be acceptable, considered the alternative of more severe illness.

### 6.2 Selectivity against Other CYPs

Whereas potency perhaps has been the driving force in previous drug discovery projects, it is only one of many features to be considered today. Most drugs interact with multiple targets, comprising several anti-targets leading to unwanted effects. To obtain selectivity for the desired target the focus has often been on reducing the effects of the drug-metabolizing CYP1, CYP2, and CYP3 enzymes.

Several of the CYP17A1 inhibitors reported to date also inhibit the drug-metabolizing CYPs, primarily CYP3A4. Due to the promiscuous nature of CYP3A4 it is not straightforward to introduce functionalities to be acceptable for the CYP17A1 enzyme but not for CYP3A4. Nevertheless, several of the previously mentioned non-steroidal CYP17A1 inhibitors are selective against CYP3A4, e.g., the stilbene analog **14** (Figure 14), **17** and **18** identified by virtual screening, **19** identified by combining the heme-binding moiety from abiraterone and galeterone, and the ketoconazole analog **20** (Figure 15).^85,89,90,93^ Unfortunately, it is not yet possible to derive some common structural denominator(s) for obtaining selectivity for CYP17A1 against CYP3A4 and the other drug-metabolizing CYPs.

Another important issue is the selectivity for CYP17A1 against CYP19A1 and CYP21A2. The steroidal inhibitors may potentially interact with all three enzymes, although it has been possible to increase CYP17A1 selective inhibition by introducing hydrogen-bonding substituents in the C6 position of abiraterone (Figure 6B).^43^ The non-steroidal inhibitor **18** (Figure 15) displayed selectivity against not only CYP3A4 but also CYP21A2, although we at present have not yet identified which of the functional groups are responsible for the observed selectivity.^89^

In a recent report, a summary of the present state-of-the-art in selectivity optimization for various CYP forms was presented.^167^ The examples comprise weakening of binding to the heme group, reduction of ligand lipophilicity, and small structural modification.

A CYP index equal to the ring count divided by the lipophilicity (cLogP) has been suggested to be a measure of the conformational rigidity corrected for the effect of lipophilicity.^168^ The authors concluded that a CYP index >2 would reduce the risk for getting compounds with submicromolar CYP3A4 binding and that use of the CYP index would increase the possibility for designing heme-binding inhibitors with reduced CYP3A4 binding.

Contrary to the above intuitive approach, a target-specific selectivity has been developed comprising the potency against the target of interest and the potency against other targets called the absolute potency and relative potency, respectively. The most selective compound was then identified by simultaneous optimization of the two potency metrics, yielding a selectivity score for the compound. The potential of this computationally more complex procedure was shown on a dataset comprising 442 kinase targets.^169^

Selectivity is clearly an unsolved issue for CYP17A1 inhibition, with the known selective compounds primarily obtained based on traditional medicinal chemistry experience and/or serendipity. Thus, more knowledge-based quantitative methods are needed to guide future design. We have not yet seen the full potential of novel methods like AI and deep learning applied to the general CYP selectivity problem nor the specific CYP17A1 selectivity problem.^170^

### 6.3 Selectivity towards Lyase Inhibition

Truly lyase-selective inhibitors would be ideal for an improved cortisol profile in humans. Generally, selectivity is always a vital factor in drug discovery targeting enzyme inhibition. In the case of CYP17A1 it is important to remember that this single enzyme is capable of catalyzing two successive reactions, a hydroxylation and a lyase transformation. The lyase inhibition is preferred over hydroxylase inhibition because this leads to a better control of circulating C19 androgen precursors without decreasing the cortisol levels and elevating ACTH.^171^ Both reactions occur in the same active site and therefore it is extremely difficult to design an inhibitor that would selectively block only one reaction. All known inhibitors act by coordinating with the heme iron, therefore by inhibiting hydroxylase reaction they will inherently affect the lyase reaction. However, some insight might be gained from substrate specificity. It is widely known that 17OH-Prog is the poor lyase substrate while 17OH-Preg is the efficient one. A structural explanation has been given pointing to different positions of these two substrates in the active site, where 17OH-Preg is positioned closer to the heme iron without making a hydrogen bond to N202.^42^ This offers a potential strategy to design inhibitors with attenuated interactions with N202.^172^ Recently, a V362M mutation found at the active site of CYP17A1 was shown to selectively decrease the lyase activity by reducing the binding of 17OH-Preg. Therefore, certain design elements could be employed for creating inhibitors that compete with 17OH-Preg for binding to the CYP17A1 active site and may have high specificity for inhibiting the CYP17A1 lyase reaction.^173^ Another important aspect of lyase selectivity is associated with cyt *b*_5_. It has been suggested that cyt *b*_5_ binding alters the CYP17A1 conformation to promote the lyase activity.^6^ This notion points to disrupting the CYP17A1-cyt *b*_5_ interaction mediated via the ternary CYP17A1-cyt *b*_5_-POR complex or even targeting cytochrome *b*_5_ itself as another potential strategy.^40,50,174^ However, conformational changes do not alter the binding of either 17OH-Preg or 17OH-Prog as measured by apparent *K*_d_ and binding kinetics.^175^ Thus, despite conformational selection appearing to be the dominant mechanism for CYP17A1 binding, structural modifications in ligand design might not easily translate to expected selectivity. Additionally, experimental structural data regarding the ternary CYP17A1-cyt *b*_5_-POR complex is also lacking.

During recent years attention has been brought to a multistep binding of lyase-selective inhibitors to CYP17A1.^73,176^ These studies indicate a rapid formation of an initial complex followed by slow conversion into the iron-complexed form. More importantly, this suggests that the formation of the heme iron heterocycle complex is not a prerequisite needed for enzyme inhibition. Consequently, iron-binding moieties may not be necessary structural features. Remembering that essentially all known inhibitors have this feature, this brings about a new generation of inhibitors that do not rely on the necessity to coordinate the heme, thus displaying potentially improved inhibition profiles.

### 6.4 Exploitation of Atoms Other than Nitrogen to Coordinate the Heme

Nearly all reported CYP17A1 inhibitors, both steroidal and non-steroidal, contain a nitrogen-containing heteroaromatic ring with the nitrogen lone-pair coordinating to the iron atom in the heme group. This bias in design of CYP17A1 inhibitors is probably inspired by the existence of azole-containing antifungals which inhibit the CYP51A1, converting lanosterol to ergosterol.^177^ The bias has also been supported by computational studies showing that a variety of nitrogen-containing heterocycles were favorable for binding to the iron in the heme group.^89,178^

The interaction between a ligand with an electron-rich nitrogen and the electron-deficient iron atom in the heme group can be considered as a classical nucleophile–electrophile interaction. The Protein Data Bank contains several examples on other nucleophilic ligands interacting with the iron atom in heme-containing proteins.

In the structure of a bacterial CYP BM3 (CYP102A1) mutant M11, the anionic form of the mercapto group in dithiothreitol coordinates to the heme group with an Fe–S distance of 2.3 Å.^179^ Several other examples of CYP structures with sulfur-containing compounds, i.e., primary thiols, coordinating the heme group are known.^179^ A handful of thioether-based nitric oxide synthase inhibitors display type II binding to the heme group. X-ray crystallography showed that the sulfur atom in some of these structures coordinated to the iron atom in the heme group with Fe–S distances of ∼2.7 Å.^180,181^

A rather unusual example of a functional group, to our knowledge not present in any drug compounds, is the alkyl isocyanide, where the carbon atom in the isocyanide group is highly nucleophilic. The structure of *n*-butyl isocyanide in complex with sperm whale myoglobin is one of several similar complexes in the Protein Data Bank, and it shows that the isocyanide carbon is located 2.1 Å above the iron atom in the heme group.^182^

Thus, based on the provided examples, we suggest that novel metal-coordinating groups should not be neglected in future design of CYP17A1 inhibitors.^183,184^

### 6.5 Physicochemical Properties

The physicochemical properties of a compound determine its absorption, distribution, metabolism, and excretion (ADME) profile and thus are vital for the success of any drug candidate. One of the key properties is compound solubility, which heavily influences the ADME profile but is also important for meaningful activities in *in vitro* assays.^185^ Factors like temperature, water content, or impurities can have significant impacts on solubility.^186^ Visual inspection of published CYP17A1 inhibitor structures suggests that many of these compounds might suffer from poor solubility. Inadequate aqueous solubility can be inferred based on the presence of dominant aromatic fragments with small numbers of polar groups. These structural features were likely put in place due to the hydrophobic nature of the enzyme binding site but also because of the facilitated synthesis. Thus, future design strategies should actively seek a careful balance between maintaining desirable activity and adequate solubility. Non-steroidal inhibitors have an inherent advantage over steroidal compounds in this regard. For instance, abiraterone, the only approved CYP17A1 inhibitor in clinical use, should be taken as a flat dose of 1000 mg administered on an empty stomach.^187^ This relatively high dose reflects poor absorption but also a very high fraction of the drug bound to the plasma proteins.^188^ The non-steroidal scaffold offers potentially greater flexibility in fine-tuning physicochemical properties.

Besides following the well-established Lipinski rule of five, tentative structural features incorporated in the molecule design can include synthesis of prodrugs, insertion of hydrophilic and ionizable groups, addition and removal of hydrogen-bonding fragments, bioisosteric replacement, and disruption of molecular symmetry and planarity.^189^ Reducing the aromatic character of a compound could potentially improve its physicochemical properties, such as solubility. The propensity to design out-of-plane substituents can enhance the compatibility between receptors and ligands. This may facilitate the creation of new protein–ligand interactions that are not attainable with a flat aromatic ring, leading to improved activity and selectivity towards a specific target and minimizing the risk of off-target effects.^190^ On the other hand, increased molecular complexity can negatively impact the readiness at which large numbers of analogs can be made.

### 6.6 Translation of *In Vitro* Effect to *In Vivo*

The translation from *in vitro* activity to *in vivo* efficacy is crucial in any drug discovery project. Many factors, absent in *in vitro* settings, determine the fate of a drug in a living organism. The high attrition rate during the drug development stage is a sobering reminder that a drug candidate can still fail despite high potency and favorable selectivity profile. Little attention is often paid to pharmacokinetics (PK) at the early drug discovery stages, while the focus is on the optimization of ligand–target interactions. The situation is especially common in academic groups. Resources available in industry drug discovery programs often permit extensive PK studies. Assessment of properties such as clearance, half-life, volume of distribution, or maximum concentration can be very costly, especially when undertaken in primate mammals.^101^ However, with CYP17A1 inhibitors, efforts should be made to measure *in vivo* testosterone levels as a predictor of potential *in vivo* efficacy. Male Sprague–Dawley rats have been successfully used to demonstrate reduction in the plasma testosterone concentration.^132,191^ Such determination should ideally include several time points. For instance, testosterone measured after 2 h and 5 h should be diminished, indicating sustained effect.^84^ Further *in vivo* assays can include profiling of other steroids. Cortisol level is of high importance because it is often dysregulated. Its imbalance during the abiraterone therapy requires concomitant administration of prednisone. Previously mentioned physicochemical properties play also important role. The most advanced next-generation non-steroidal CYP17A1 inhibitors, such as orteronel and seviteronel, described above, exhibit good bioavailability and general ADME properties in addition to an improved selectivity towards the desired inhibition of the lyase activity of the CYP17A1 enzyme. Both these compounds were taken into clinical development. Unfortunately, orteronel was discontinued after phase III as it failed to extend overall survival rates in the target metastatic, hormone-refractory prostate cancer patient group. Seviteronel is still in clinical development although issues with drug-related tolerability in a phase 2 trial have been reported.

### 6.7 Emerging Competing Prostate Cancer Therapies

Prostate cancer therapy has been at the forefront of CYP17A1 inhibitor development. Numerous innovative treatment alternatives for prostate cancer have emerged in recent years. Histone deacetylase (HDAC) inhibitors, such as vorinostat, pracinostat, panobinostat, and romidepsin, serve as notable examples. Although all four underwent clinical trials, they ultimately fell short due to a majority of patients experiencing toxicity or disease progression. At present, two critical pieces of information are missing from studies on HDAC inhibitors in cancer: first, the expression profiles of various HDACs in prostate cancer models, and second, the involvement of AR with HDACs in prostate cancer.^192^

Recently, aberrant fatty acid activation of peroxisome proliferator-activated receptors (PPARs) resulting from dysregulated lipid signaling has been implicated as a crucial factor in prostate cancer. Fatty-acid-binding proteins (FABPs), particularly FABP5, facilitate PPAR activation. FABP5 is overexpressed in prostate cancer and is associated with poor patient prognosis and survival.^193^ However, the identification of FABP5 as a molecular target for prostate cancer remains in its early stage, with several challenges to overcome, primarily due to the ubiquity of FABP5.

A substantial number of DNA-damage repair (DDR) pathways have been found to be frequently dysregulated in advanced prostate cancer stages. Tumors with compromised ability to repair double-strand DNA breaks via homologous recombination are highly sensitive to the inhibition of poly(ADP) ribose polymerase (PARP) enzyme. Olaparib was the first agent to show benefit in patients with DDR-deficient prostate cancer.^194^

Proteolysis-targeting chimeras (PROTACs) exemplify the therapeutic approach of induced protein degradation. These heterobifunctional molecules create a trimeric complex between a target protein and an E3 ubiquitin ligase, facilitating target ubiquitination and subsequent degradation. AR-targeting PROTACs, notably ARCC-4 (an enzalutamide-based von Hippel–Lindau (VHL)-recruiting AR PROTAC), have been proposed and shown to be superior to enzalutamide.^195^ The primary advantages include inducing apoptosis and inhibiting the proliferation of AR-amplified prostate cancer cells, as well as effectively degrading clinically relevant AR mutants associated with antiandrogen therapy. PROTAC-mediated AR degradation could potentially address several AR-dependent drug resistance mechanisms characteristic of castration-resistant prostate cancer.

When comparing these emerging therapies to CYP17A1 inhibition, it is crucial to consider that, at the molecular level, prostate cancer is primarily driven by excessive signaling via the androgenic pathway. While androgenic signaling is considered the primary driver of CRPC, androgen-independent signaling pathways might also contribute to CRPC progression.^196^ Nonetheless, as CRPC advances, the concentration of PSA continues to rise. Since PSA is regulated by androgenic signaling, this implies that androgenic signaling remains involved in CRPC progression.^197^ Therefore, androgen signaling remains central to prostate cancer pharmacology, leaving CYP17A1 inhibition as an important and attractive strategy.

As the field of prostate cancer therapeutics continues to evolve, it is possible that the ongoing research into the interplay between these pathways may uncover additional synergies and opportunities for combination therapies, further enhancing the effectiveness of cancer treatment.

### 6.8 Concluding Remarks

In conclusion, there is still a need for improved compounds as potent and selective inhibitors of the steroidogenic CYP17A1 enzyme as a target for treatment of serious hormone-dependent cancer diseases, e.g., prostate cancer. The extensive work in recent years has provided compounds, belonging to the non-steroidal class, with improved activity and selectivity as well as translational properties. However, none of these has yet reached clinical practice. As outlined above, there are key features that still need to be addressed for the next-generation compounds, such as selectivity towards other CYPs, specificity for the CYP17A1 lyase inhibition, and acceptable physicochemical properties. We hope that these issues can be solved with medicinal chemistry efforts towards the optimal compound.

## Supporting Information Available

Three-dimensional model of CYP17A1-cyt b5 complex anchored in the membrane, available as an animation illustrating the 3D relationships of the structure and interactions (MP4)

## Abbreviations Used

17OH-Preg: 17α-hydroxypregnenolone
17OH-Prog: 17α-hydroxyprogesterone
AR: androgen receptor
ADT: androgen deprivation therapy
CoMFA: comparative molecular field analysis
CRPC: castration-resistant prostate cancer
cyt b5: cytochrome b5
CYP17A1: cytochrome P450 17A1
DDR: DNA damage repair
DHEA: dehydroepiandrosterone
DFT: density functional theory
DHT: dihydrotestosterone
GC: gas chromatography
HDAC: histone deacetylase
HPLC: high-performance liquid chromatography
MS: mass spectrometry
PARP: poly(ADP) ribose polymerase
PPAR: peroxisome proliferator-activated receptor
PK: pharmacokinetics
POR: P450 reductase
PCOS: polycystic ovary syndrome
PROTAC: proteolysis targeting chimera
PSA: prostate-specific antigen
RIA: radioimmunoassay
SOSA: optimization of side activities
TLC: thin-layer chromatography
